# Effects of Fiber Reinforcement on Clay Aerogel Composites

**DOI:** 10.3390/ma8085258

**Published:** 2015-08-21

**Authors:** Katherine A. Finlay, Matthew D. Gawryla, David A. Schiraldi

**Affiliations:** Department of Macromolecular Science and Engineering, Case Western Reserve University, Cleveland, OH 44106-7202, USA; E-Mails: katfin@gmail.com (K.A.F.); matt.gawryla@owenscorning.com (M.D.G.)

**Keywords:** clay, aerogel, poly(vinyl alcohol), fibers, composite, mechanical properties

## Abstract

Novel, low density structures which combine biologically-based fibers with clay aerogels are produced in an environmentally benign manner using water as solvent, and no additional processing chemicals. Three different reinforcing fibers, silk, soy silk, and hemp, are evaluated in combination with poly(vinyl alcohol) matrix polymer combined with montmorillonite clay. The mechanical properties of the aerogels are demonstrated to increase with reinforcing fiber length, in each case limited by a critical fiber length, beyond which mechanical properties decline due to maldistribution of filler, and disruption of the aerogel structure. Rather than the classical model for reinforced composite properties, the chemical compatibility of reinforcing fibers with the polymer/clay matrix dominated mechanical performance, along with the tendencies of the fibers to kink under compression.

## 1. Introduction

Aerogels are materials that are produced from wet gels by replacement of solvent with air, typically exhibiting bulk densities of 0.1 g/cm^3^ or lower. The preparation of inorganic aerogels was first described by Kissler, who slowly exchanged the water in aqueous silica with volatile organic solvents, which, upon removal, produced low density solids [1,2]. Great progress has been made in the production and property enhancement of silica aerogels in recent years [3,4,5]. A similar process was described for the conversion of aqueous clay gels into a fabric-like solid first by MacKenzie [6], with a “house of cards” structure then proposed by Van Olphen and others [7,8]. In our own work, we have demonstrated a robust freeze-drying process for converting common smectite clays, such as sodium montmorillonite and bentonite, into aerogels with bulk densities in the range of 0.02–0.10 g/cm^3^ [9]. These clay aerogel materials are fragile, exhibiting mechanical properties similar to those of balls of cotton fiber, and hence are easily crushed or irreversibly damaged under low stress levels. Introduction of a wide variety of matrix polymers, including biologically-derived proteins and polysaccharides, can convert the aerogels into more mechanically-robust composites which are reminiscent of polymer foams [10,11,12,13,14,15,16], which can be further modified by directional freezing of the aqueous clay/polymer composition [12]. Such polymer/clay aerogel composites can possess excellent thermal insulation properties [17], and can be modified to be electrically conductive [18], highly absorbant [19], or to exhibit environmentally responsive properties [20]. Further physical modifications which can substantially enhance the physical properties of polymer/clay aerogel composites include biomimetic mineralization [21], incorporation of rigid nanowhiskers [22], and reinforcement with fibers [23]. These aerogels can also be incorporated into traditional, fabric-reinforced composite structures, benefiting from adhesive penetration of both fabric and aerogel to produce materials whose mechanical properties and densities are reminiscent of balsa or cork [24]. In a preliminary study we reported that the combination of biologically-based fibers, domestic clay, and polymers (especially those which are bio-based and/or biodegradable) can produce environmentally-benign materials with substantially improved mechanical properties such that they are suitable for a wide range of end uses [25]. Exploration of the effects of reinforcing fiber length and composition on mechanical properties is reported in the present work.

## 2. Experimental Section

### 2.1. Materials

Sodium Montmorillonite (PGW, Nanocor, Abertdeen, MS, USA), poly(vinyl alcohol) (PVOH, with a number average molecular weight (*M*_n_) of 108,000 kD, a polydispersity index (PDI) of ~1.7, and a hydrolyzation of 99.7% from Polysciences, Warrington, PA, USA) was used as received. PVOH Fibers (RECS15, 8 mm length, Kuraray Americas, Houston, TX, USA) were used as received. Hemp and silk fibers (mulberry silk produced by Bombyx Mori, Aurora Silk, Portland, OR, USA) and soy silk (Meilke’s Farm LLC, Mauston, WI, USA) were used as received except for cutting to length. Deionized water was prepared using a Barnstead ROpure low-pressure, reverse osmosis system.

### 2.2. Typical Aerogel Preparation

In a 100 mL beaker, 25 mL of a 5 w/v % PVOH solution was slowly added to a 10 w/v % clay gel and mixed thoroughly by hand. Fibers of the appropriate length (2–20 mm) were then added at a 1 w/v % concentration, and mixing was completed with a hand blender. The resulting clay/fiber hydrogel mixtures were poured into four 5 dram cylindrical polystyrene vials (Fisher Scientific). These forms were immediately placed into an ethanol/solid carbon dioxide bath to freeze their contents. Once the samples were completely frozen, they were removed from the ethanol/dry ice bath, and placed in a freezer at −12 °C for storage. Samples were then placed in a VirTis AdVantage^®^ EL-85 freeze-dryer (SP Scientific, Warminster, PA, USA), where high vacuum (3 μbar/25 °C) was applied to sublime the ice and reveal the aerogel structures of the samples. Cylindrical test pieces for compression testing, measuring approximately 20 mm in both height and diameter, were cut from each sample using a band saw. 

### 2.3. Characterization 

Compression testing was conducted on the prepared test pieces (measuring 20 mm height and 20 mm diameter) using an Instron model 5565 universal testing machine fitted with a 1 kN load cell. These tests were performed at a constant strain rate of 1 mm/min and were stopped when the 1 kN load limit (machine limit) was reached; three samples from three separate batches were tested for each composition of interest, resulting in nine total test samples. All of the samples in this study were sufficiently robust to be handled during testing. 

### 2.4. Analysis

Density values were calculated from the masses and initial dimensions of compression test pieces. For each polymer loading, density values were averaged, and the standard deviation and the standard error were calculated. The load-displacement data from each compression test were converted into stress-strain data, and a modulus was calculated from the linear-elastic region of the stress strain curve. For each fiber length, modulus values were averaged, and the standard deviation was calculated. 

## 3. Results and Discussion

**Figure 1:**
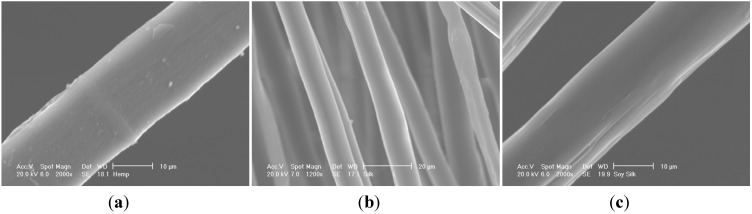
Hemp (**a**), silk (**b**) and soy silk fibers (**c**) used in this study.

Freeze drying of aqueous clay/PVOH/fiber samples readily produced low density, fiber-reinforced aerogel composite materials, as we have reported previously [25]. In that preliminary work we proposed that the fiber/clay aerogel structures appear to be almost woven, with clay layers in the “warp” direction, and reinforcing fibers in the “weft”. Images of the specific reinforcing fibers used in this study, hemp (diameter 20 ± 2 μm) , silk (diameter 10 ± 2 μm) and soy silk (diameter 20 ± 2 μm), are given in Figure 1. Samples for compression testing were produced using standard polystyrene cylindrical vials as their molds; all sides of the resultant test parts were smooth with the exception of the top which exhibited the rough texture typical of ice cube tops. The tops of the cylindrical samples were leveled by cutting to size with a band saw. Density measurements for the composites produced from solutions containing 5 wt % clay, 5 wt % PVOH, and 1 wt % of the various fibers cut to the indicated lengths are given in Table 1, Table 2 and Table 3. The densities of the fiber-reinforced samples tended to increase slightly with increasing fiber lengths at constant fiber loadings. Such a densification requires a decrease in the interlayer gallery spacing present in the “house of cards” aerogel structures. 

**Figure 2:**
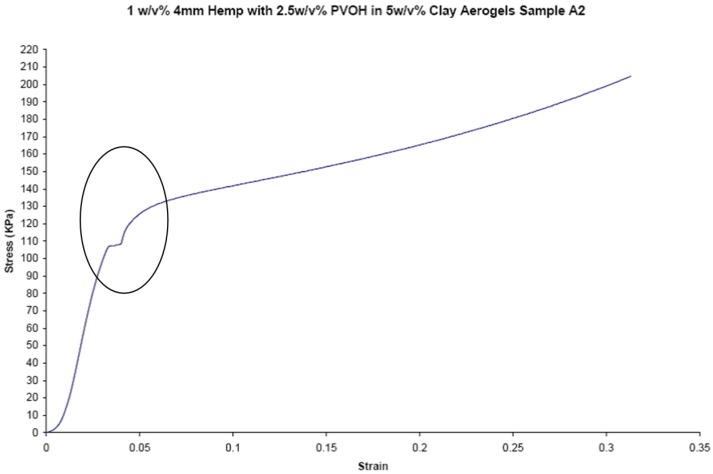
Stress-strain curve for a hemp sample, circled portion shows delamination plateau.

Compressive moduli and compressive strengths for the three series of fiber reinforced aerogel composites are given in Table 4, Table 5 and Table 6. These data show that silk and soy silk produced higher compressive moduli and strengths than did hemp. Adjusting for density the specific moduli of soy silk-reinforced samples, 8250 kPa·cm^3^/g at 2 mm fiber length, exceeded those of silk-reinforced, 7200 kPa·cm^3^/g at 2 mm fiber length, and especially those of the hemp-reinforced samples, 5570 kPa·cm^3^/g at 2 mm fiber length, produced in this study. Poor adhesion between the matrix and the hemp fiber is likely a major reason for the reduced performance of this reinforcing fiber in the present system. The stress-strain curves for a typical hemp-containing sample (Figure 2) show a small inflection point or plateau, just above 100 kPa. This plateau is attributed to the delamination of the fiber from the matrix. This delamination is not seen in any of the silk or soy silk stress-strain curves. It is therefore believed that the good adhesion between the matrix and soy silk and silk fibers, and the poor adhesion between hemp and the matrix, can likely be attributed to fiber polarities and the ability of these materials to hydrogen bond to the polar PVOH and clay. Hemp, a composite of cellulose and hydrophobic lignin, would not be expected to interact well with clay and the matrix polymer. While cellulose hydrogen bonds well to itself, it has been shown to be a poor matrix for polymer/clay aerogels [26]. Lignin was recently shown to be an equally poor matrix for polymer/clay aerogels [27]. However, the protein-based fibers, silk and soy silk, can be readily expected to hydrogen bond to both montmorillonite clay and the poly(vinyl alcohol) polymer matrix.

**Figure 3:**
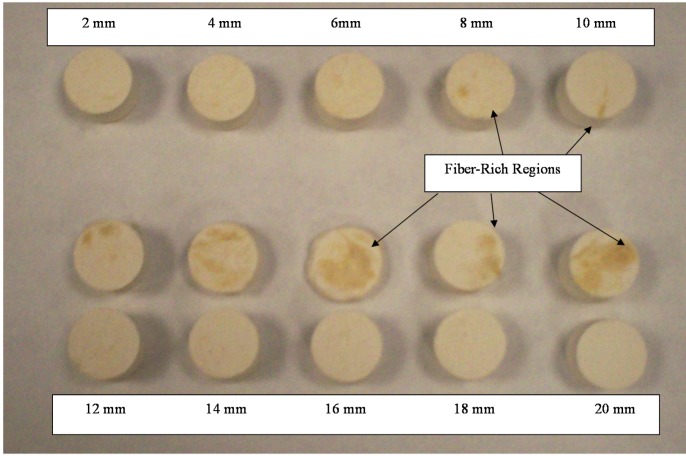
Cross sections of soy silk samples, 2–20 mm fiber lengths, uncut samples in bottom row.

Fiber length appears to strongly affect the mechanical properties of the aerogel composites. A sharp decrease in mechanical properties can be observed with reinforcing fiber lengths over 6 mm. The trend with hemp was less well-defined, though it is directionally similar with a maximum value perhaps at 14 mm. Examining the distribution of fibers within the aerogel composite matrix, Figure 3, it can be observed that as fiber lengths increase beyond a critical length, the homogeneity of fiber distribution is lost. This lack of homogeneity correlates well with the declining mechanical properties of samples loaded with longer fiber lengths. A critical fiber length, *l*_c_, that is independent of the applied stress is defined as the minimum fiber length where the fiber ultimate strength, σ_fu_, can be reached. The critical fiber length can be calculated according to Equation (1) which relies upon the fiber ultimate stress, the shear stress at the fiber-matrix interface, τ_i_, (which is assumed to be equal to the matrix shear yield strength), and the fiber diameter, *d*_f_ [28]. Making use of the fiber and matrix values given in Table 6, critical fiber lengths of 56, 4.8 and 4.0 mm can be calculated for hemp, silk, and soy silk respectively. Thus, the experimental data for silk and soy silk materials appear to be in good agreement with the theoretical critical length calculations, whereas for hemp, a significant deviation from theory exists. 

**Table 1:** Average densities for each fiber length of the hemp compression series samples.

Fiber Length (mm)	N	Density (g/cm^3^)
2	9	0.070 ± 0.004
4	9	0.069 ± 0.002
6	9	0.068 ± 0.004
8	9	0.069 ± 0.007
10	9	0.070 ± 0.004
12	9	0.080 ± 0.010
14	9	0.080 ± 0.010
16	9	0.077 ± 0.009
18	9	0.080 ± 0.007
20	9	0.080 ± 0.010

**Table 2:** Average densities for each fiber length of the silk compression series samples.

Fiber Length (mm)	N	Density (g/cm^3^)
2	9	0.075 ± 0.002
4	9	0.078 ± 0.004
6	9	0.077 ± 0.007
8	9	0.076 ± 0.005
10	9	0.090 ± 0.010
12	9	0.090 ± 0.007
14	9	0.080 ± 0.010
16	9	0.080 ± 0.008
18	9	0.082 ± 0.010
20	9	0.080 ± 0.020

**Table 3:** Average densities for each fiber length of the soy silk compression series samples.

Fiber Length (mm)	N	Density (g/cm^3^)
2	9	0.081 ± 0.003
4	9	0.081 ± 0.002
6	9	0.082 ± 0.002
8	9	0.080 ± 0.002
10	9	0.078 ± 0.003
12	9	0.070 ± 0.030
14	9	0.070 ± 0.020
16	9	0.080 ± 0.020
18	9	0.080 ± 0.010
20	9	0.090 ± 0.020

**Table 4:** Compressive moduli and yield strengths *vs.* fiber length for the hemp compression series.

Fiber Length (mm)	N	Compressive Modulus (kPa)	Compressive strength at yield (kPa)
2	8	3900 ± 700	100 ± 10
4	8	4700 ± 700	110 ± 20
6	9	5000 ± 3000	80 ± 30
8	9	6000 ± 2000	100 ± 40
10	8	3700 ± 800	100 ± 30
12	8	5000 ± 1000	90 ± 30
14	9	6000 ± 3000	100 ± 40
16	9	5000 ± 2000	100 ± 40
18	9	4000 ± 1000	110 ± 40
20	8	5000 ± 2000	80 ± 30

**Table 5:** Compressive moduli and yield strengths *vs.* fiber length for the silk compression series.

Fiber Length (mm)	N	Compressive Modulus (kPa)	Compressive Strength at Yield (kPa)
2	9	5000 ± 1000	180 ± 20
4	8	7000 ± 1000	190 ± 10
6	7	6900 ± 400	190 ± 10
8	9	6000 ± 1000	180 ± 10
10	9	4200 ± 100	130 ± 20
12	9	3700 ± 900	100 ± 10
14	9	4000 ± 1000	100 ± 30
16	8	5000 ± 2000	130 ± 60
18	8	4000 ± 800	130 ± 30
20	9	5000 ± 3000	100 ± 40

**Table 6:** Compressive moduli and yield strengths *vs.* fiber length for the soy silk compression series.

Fiber Length (mm)	N	Compressive Modulus (kPa)	Compressive Strength at Yield (kPa)
2	9	6600 ± 600	220 ± 10
4	9	8000 ± 1000	240 ± 20
6	9	7500 ± 800	240 ± 10
8	9	7000 ± 1000	220 ± 20
10	9	6700 ± 900	200 ± 120
12	9	6000 ± 1000	190 ± 10
14	9	6000 ± 1000	170 ± 20
16	9	6000 ± 2000	190 ± 10
18	9	6000 ± 2000	180 ± 50
20	9	6000 ± 1000	160 ± 30

It was shown by Thomason that the composite strength is impacted negatively with increasing fiber diameter, and these effects are increased further when there is a large distribution of fiber diameters as opposed to one single diameter [28]. In addition, once the fiber has debonded from the matrix partially a small flaw is created within the composite that is proportional to the size of the fiber diameter. A larger diameter results in a larger flaw and as such a reduced composite strength [29]. The hemp fibers used in this study had a large average diameter of 20 μm, and varies due to the retting process used in manufacturing. It is therefore believed that the large fiber diameter and distribution of diameters found in hemp fiber will likely reduce the strength of the composite, and contribute to the deviations from theory observed in this work for the hemp fiber system.

(1) $$l_{\text{c}}=\frac{{\text{σ}}_{\text{fu}}}{2{\text{τ}}_{\text{i}}}d_{\text{f}}$$

Mechanical property data for the three reinforcing fiber materials examined in this study, along with estimated properties of polymer/clay aerogels under shear conditions, are given in Table 7. Using the Halpin-Tsai equations the stiffness of a discontinuous fiber-reinforced composite can be calculated. These equations will only calculate the in-plane stiffness of a single, transversely isotropic sheet that has all the fibers unidirectionally aligned in the plane of the sheet. Several assumptions are made in this theory, first that both the filler and the matrix are homogenous and linearly elastic, second, that the filler and the matrix are void-free, third, that there is perfect contact between the filler and the matrix at the interface, and fourth, that the filler is perfectly dispersed. Use of Halpin-Tsai in such a “house of cards” structure is perhaps a stretch, but since the aerogel structure is constructed of composite elements, this analysis is worthy of consideration. The equations are:

(2) $$\frac{\bar{P}}{P_{\text{m}}}=\frac{1+\text{ζη}V_{\text{f}}}{1-\text{η}V_{\text{f}}}$$

(3) $${\text{ν}}_{12}={\text{ν}}_{\text{f}}V_{\text{f}}+{\text{ν}}_{\text{m}}V_{\text{m}}$$

(4) $$\text{η}=\frac{\left(\frac{P_{\text{f}}}{P_{\text{m}}}-1\right)}{\left(\frac{P_{\text{f}}}{P_{\text{m}}}+\text{ζ}\right)}$$

where Equation (3) represents the in-plane Poisson ratio and *V*_f_ is the fiber volume fraction, $\bar{P}$ is the property of the composite, *P*_m_ is the property of the matrix, ξ is the geometry factor, η is the efficiency factor, ν_f_ is the volume fraction of filler, ν_m_ is the volume fraction of matrix, *P* is the engineering tensile stiffness parallel (*E*_11_) or perpendicular (*E*_22_) to the fiber direction or the in-plane shear stiffness (*G*_12_), and all subscripts f refer to fiber and m refer to matrix. *P*_f_ refers to either the modulus of the fiber (*E*_f_) or the shear stiffness of the fiber (*G*_f_). *P*_f_ can also be replaced by *P*_m_ which can be equal to either the modulus of the matrix (*E*_m_) or the shear stiffness of the modulus (*G*_m_). Reliable estimates of the factor are:

(5) $$\begin{array}{l} {\text{ζ}}_{E_{\text{11}}}=2\frac{l}{t},\\ {\text{ζ}}_{E_{\text{22}}}=2\frac{l}{w},\\ {\text{ζ}}_{G_{\text{12}}}={\left(\frac{w}{t}\right)}^{\text{1.732}} \end{array}$$

where *l*, *w*, and *t* are the length, width, and thickness of the reinforcement of the composite. For a circular fiber reinforcement l is the length of the fiber and *t* and *w* are both equivalent to the diameter of the fiber. Therefore, is always equal to 1 for all fiber reinforcement and the factor can be rewritten in terms of the aspect ratio (*l*/*d*) [27]:

(6) $$\begin{array}{l} {\text{ζ}}_{E_{\text{11}}}=2\frac{l}{d},\\ {\text{ζ}}_{E_{\text{22}}}=2\frac{l}{d},\\ {\text{ζ}}_{G_{\text{12}}}=1 \end{array}$$

**Figure 4:**
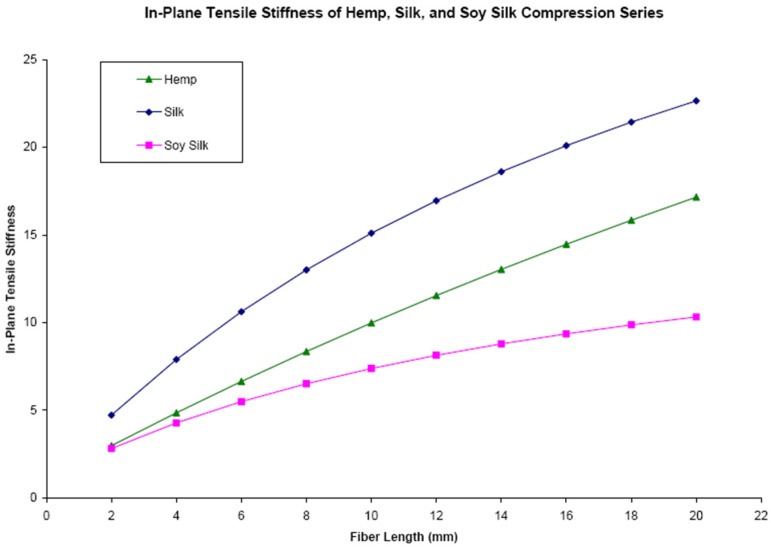
In-plane tensile stiffness *vs.* the fiber length for the soy silk, silk, and hemp compression series (purple—soy silk, blue—silk, green—hemp).

Comparing all three fiber sets, soy silk has the largest densities, moduli, and strengths in the 2–10 mm fiber length range while hemp has the smallest values (Table 1, Table 2, Table 3, Table 4, Table 5 and Table 6). However, these measured strengths of the fiber reinforced aerogels are not in good agreement with the theoretical values calculated with the Halpin-Tsai theory. Using the in-plane stiffness, σ_cu_, as calculated using Equations (2)–(5) and values from Table 7, it is seen in Figure 4, that with increasing fiber length the stiffness of the composites are expected to increase. However, the Halpin-Tsai equations assume that the fibers are distributed perfectly throughout the matrix, and as discussed previously and shown in Figure 3, the fibers are not evenly distributed after 6 mm. Thus, these theoretical values cannot be considered as an accurate representation of the composites after a length of 6 mm. 

The theory also predicts the silk samples are the stiffest, followed by hemp and soy silk, but this is also not in agreement with the collected data. This can likely be attributed to the Halpin-Tsai equations only taking into account the modulus and diameter of the fiber and matrix, and the assumption of perfect adhesion between the fiber and the matrix. However as stated previously, hemp does not have strong adhesion in the matrix and in addition, both silk and hemp are highly susceptible to moisture absorption and kink band defects. Moisture absorption could lead to a changing fiber diameter, which could result in fiber delamination from the matrix, and kink band defects can lead to mirobuckles. None of these properties are accounted for in the Halpin Tsai calculations, and as such the collected data does not agree with the theoretical values. Calculations of the compressive strength were also performed to determine if the main mechanism of failure was via the extensional mode or the shear mode. A compressive strength of 494 kPa for hemp, 380 kPa for silk, and 230 kPa for soy silk were calculated using Equation (8) for the extensional mode, whereas a compressive strength of 1.95 MPa was calculated for all composites using Equation (7) for the shear mode. This is because the shear failure mode is only dependent upon the shear modulus of the matrix, not the filler. 

Shear Mode:

(7) $$\begin{array}{l} {\text{σ}}_{\text{cu}}\approx \frac{G_{\text{m}}}{\left(1-V_{f}\right)},\\ {\text{σ}}_{\text{cu}}\approx \frac{1930000}{\left(1-0.01\right)},\\ {\text{σ}}_{\text{cu}}\approx 1.95\text{MPa} \end{array}$$

Extentional Mode, Hemp:

(8) $$\begin{array}{l} {\text{σ}}_{\text{cu}}\approx 2V_{\text{f}}{\left[\frac{V_{\text{f}}E_{\text{m}}E_{\text{f}}}{3\left(1-V_{\text{f}}\right)}\right]}^{\frac{1}{2}},\\ {\text{σ}}_{\text{cu}}\approx 2\left(0.01\right){\left[\frac{\left(0.01)(0.015\times 10^{9})(12.1\times 10^{9}\right)}{3\left(1-(0.01)\right)}\right]}^{\frac{1}{2}},\\ {\text{σ}}_{\text{cu}}\approx 494\text{KPa} \end{array}$$

Both equations overestimate the measured compressive strengths of these composites, provided in Table 4, Table 5 and Table 6, except for the strength of soy silk which agrees well with the extensional mode strength calculation. The severe overestimation of the compressive strength by the shear mode and the smaller overestimation of the strength of hemp and silk reinforced composites by the extensional mode suggest that the primary mode of failure in these composites is due to the extensional mode. This failure mode characterized by the microbuckling at fiber kink bands in localized areas of the composite [27]. As discussed previously, hemp and silk fibers are highly susceptible to kink bands, consistent with failure of these composites via the extensional mode.

**Table 7:** Properties of hemp, silk, and soy silk fiber, and poly(vinyl alcohol) (PVOH)/clay aerogel. The shear modulus and shear strength were taken to be equal to those of expanded polystyrene with the same density.

Property	Hemp Fiber	Silk Fiber	Soy Silk Fiber	PVOH/Clay Aerogel
Strain Elongation (%)	1.6	14	18	-
Tensile Strength (MPa)	690	120	5	-
Elastic Modulus (GPa)	12.1	7	2.6	0.015
Density (g/cm^3^)	-	1.34	1.29	0.05
Shear modulus (MPa)	-	-	-	1.93
Shear Strength (kPa)	-	-	-	124

## 4. Conclusions

Novel, low density structures which combine biologically-based fibers with clay aerogels were produced in an environmentally benign manner using water as solvent, and no additional processing chemicals. A comparison of three different reinforcing fibers, silk, soy silk, and hemp, showed that mechanical properties of the aerogels increase with reinforcing fiber length, as expected, however each material exhibits a critical fiber length, beyond which mechanical properties decline due to maldistribution of filler, and disruption of the aerogel structure. This critical fiber length agreed well with a theoretical prediction for silk and soy silk fibers, and is believed to be inaccurate for hemp due to the large and varying diameter size. In addition, the Halpin Tsai equations were shown to be poor predictors of mechanical properties for these materials, as the chemical compatibility of reinforcing fibers with the polymer/clay matrix dominated mechanical performance, along with the tendencies of the fibers to kink under compression. The knowledge gained from this work will be valuable going forward as such low density aerogel materials find applications in packaging and reinforcement, allowing optimized reinforcement of such products.
